# The Raf-1 inhibitor GW5074 and dexamethasone suppress sidestream smoke-induced airway hyperresponsiveness in mice

**DOI:** 10.1186/1465-9921-9-71

**Published:** 2008-11-03

**Authors:** Ying Lei, Yong-Xiao Cao, Cang-Bao Xu, Yaping Zhang

**Affiliations:** 1 Department of Pharmacology, Xi'an Jiaotong University College of Medicine, No. 76, Yanta West Road, Xi'an, Shaanxi Province 710061, PR China; 2 Division of Experimental Vascular Research, Institute of Clinical Science in Lund, Lund University, Lund, Sweden

## Abstract

**Background:**

Sidestream smoke is closely associated with airway inflammation and hyperreactivity. The present study was designed to investigate if the Raf-1 inhibitor GW5074 and the anti-inflammatory drug dexamethasone suppress airway hyperreactivity in a mouse model of sidestream smoke exposure.

**Methods:**

Mice were repeatedly exposed to smoke from four cigarettes each day for four weeks. After the first week of the smoke exposure, the mice received either dexamethasone intraperitoneally every other day or GW5074 intraperitoneally every day for three weeks. The tone of the tracheal ring segments was recorded with a myograph system and concentration-response curves were obtained by cumulative administration of agonists. Histopathology was examined by light microscopy.

**Results:**

Four weeks of exposure to cigarette smoke significantly increased the mouse airway contractile response to carbachol, endothelin-1 and potassium. Intraperitoneal administration of GW5074 or dexamethasone significantly suppressed the enhanced airway contractile responses, while airway epithelium-dependent relaxation was not affected. In addition, the smoke-induced infiltration of inflammatory cells and mucous gland hypertrophy were attenuated by the administration of GW5074 or dexamethasone.

**Conclusion:**

Sidestream smoke induces airway contractile hyperresponsiveness. Inhibition of Raf-1 activity and airway inflammation suppresses smoking-associated airway hyperresponsiveness.

## Background

Airway hyperreactivity is the major feature of asthma and chronic airway inflammation. Sidestream smoke is a strong risk factor for asthma and chronic airway inflammation[1]. Epidemiologic studies have revealed that exposure to environmental cigarette smoke exacerbates airway hyperreactivity in asthma and chronic airway inflammation with increased symptom severity, greater frequencies of medication usage, and more emergency room visits [2]. There are close relationships between smoking, airway inflammation and hyperreactivity. Inhibition of airway inflammatory signaling may improve smoking-associated airway inflammation and hyperresponsiveness.

Dysfunction and/or damage to airway epithelium and smooth muscle cells by mainstream and sidestream smoke result in airway inflammation and hyperreactivity. Using an *in vitro *model, we demonstrated that exposure to smoke particles [3] or cytokines (TNF-α and IL-1β) [4,5] induces airway hyperresponsiveness through up-regulation of the G-protein coupled receptors (GPCRs) for bradykinin and endothelin. Activation of intracellular mitogen-activated protein kinase (MAPK) inflammatory signal transduction pathways are responsible for the up-regulation of GPCRs in the airway [5,6]. As one of the three members in the Raf family, Raf-1 (C-Raf) is the most widely expressed. It is the initial and key protein kinase in the MAPK signal transduction cascade [7]. Transient activation of Raf-1 results in changes in smooth muscle cell functions, such as proliferation, whereas sustained activation results in differentiation through the regulation of various ERK substrates [8,9]. The Raf-1 inhibitor GW5074 was used in the present investigation to determine if the Raf/MAPK signaling pathway is involved in sidestream smoke-induced airway inflammation and hyperreactivity.

Cigarette smoke exposure is a strong risk factor for airway inflammation and hyperreactivity. However, the underlying molecular mechanisms by which smoke leads to airway damage are still elusive. In the present study, use of an *in vivo *model of sidestream smoke exposure revealed that mice exposed to sidestream smoke exhibit airway inflammation and hyperreactivity. Dexamethasone and a Raf-1 inhibitor are both able to suppress smoke-induced airway inflammation and hyperreactivity.

## Methods

### Mice and reagents

Six-week-old male ICR mice were purchased from the Animal Center of Xi'an Jiaotong University College of Medicine and maintained on normal diet, with free access to food and water. The housing facility was maintained at 20–22°C and 60%–80% relative humidity. After one week in a quarantine room, the mice were used for the experiments. GW5074 was a gift from Professor Yuhai Tang at the Science College of Xi'an Jiaotong University, China. Dexamethasone, carbachol, isoprenaline and indomethacin, were purchased from Sigma (St. Louis, U.S.A). Sarafotoxin 6c and endothelin-1 were purchased from Auspep (Parkville, Australia).

### Sidestream smoke exposure and experimental protocol

The mice were randomly divided into six groups: (1) fresh air exposure + sham; (2) sidestream smoke exposure + sham; (3) sidestream smoke exposure + dexamethasone 1 mg/kg; (4) sidestream smoke exposure + dexamethasone 0.3 mg/kg; (5) sidestream smoke exposure + GW5074 2 mg/kg; (6) sidestream smoke exposure + GW5074 0.5 mg/kg. The used dosages of dexamethasone [10-13] and GW5074 [14] were based on previous studies using an *in vivo *mouse model.

Sidestream smoke is defined as the smoke emitted from the tip of a smoking cigarette [15]. The cigarette smoke in the present setup was generated from the lit end of a cigarette; therefore, the mice in this study were exposed to sidestream cigarette smoke. Exposure of the mice to sidestream smoke was performed in a whole-body, 0.108 m^3 ^(18 cm × 25 cm × 24 cm) plastic exposure chamber, maintained at 21 ± 1°C and 40% ± 5% relative humidity. The cigarette smoke was generated from commercially-available filter cigarettes (Marlboro, 1.0 mg of nicotine and 12 mg of tar). Twenty mice were put in the chamber and each cigarette was lit on the end intended to be lit and allowed to freely burn for 15 min while resting on the stainless wire netting above the animals in the chamber. Then, the cigarette smoke was held in the chamber for another 25 min. Fresh air inhalation was performed for 10 min after every 40 min of sidestream smoke exposure.

The mice were repeatedly exposed to the smoke of four cigarettes (or fresh air) each day on six consecutive days per week for four weeks under the same conditions. After the first week of smoke exposure, dexamethasone was administrated intraperitoneally every other day and GW5074 was administrated intraperitoneally every day for three weeks. The same volume of saline was used as a sham control. The experimental protocols for using mice have been reviewed and approved by the animal ethics committee at Xi'an Jiaotong University.

### Trachea ring segment myograph

Twenty-four hours after the last cigarette smoke or room air exposure, the mice were sacrificed by cervical dislocation and the whole trachea was removed gently. The trachea was then dissected free of adhering tissue under a microscope and cut into three or four segments, each with three cartilages per ring. The segments were immersed into tissue baths containing 1 mL of Kreb's solution (mM/L: NaCl 119, NaHCO_3 _15, KCl 4.6, CaCl_2 _1.5, NaH_2_PO_4_ 1.2, MgCl_2 _1.2, glucose 5.6). The solution was continuously equilibrated with 5% CO_2 _in O_2 _to result in a stable pH of 7.4. Each tracheal segment was mounted on two L-shaped metal prongs. One prong was connected to a force-displacement transducer for continuous recording of isometric tension by the Chart software. Another prong was connected to a displacement device, allowing adjustment of the distance between the two parallel prongs. Following equilibration, a pre-tension of about 2 mN was applied to each segment and adjusted to this level of tension for at least 1 h. The segments were contracted with 60 mM potassium chloride to test the contractile function. To inhibit epithelial prostaglandin release, the segments were incubated with 3 mM indomethacin[16,17] 30 min before administration of sarafotoxin 6c and endothelin-1.

Concentration-contraction curves of the trachea ring segments were obtained by cumulatively administration of potassium chloride (30, 60, 90 mM), carbachol (10^-8^-10^-4 ^M), sarafotoxin 6c (10^-10^-10^-7 ^M) and endothelin-1 (10^-10^-10^-7 ^M), respectively. To study endothelin ET_A _receptor-mediated contractions, the experiment started with the desensitization of the ET_B _receptors by inducing a concentration response curve to sarafotoxin 6c. When the maximal contraction by sarafotoxin 6c was reached, it was allowed a fade away until the contractile curves fell to baseline level, which was considered as a total desensitization[18,19]. To study the dilation effect of a β-adrenoceptor agonist, a sustained pre-contraction was obtained by using 2 × 10^-7^ M carbachol, and subsequently, cumulative administration of the β-adrenoceptor agonist, isoprenaline, was added to the baths to induce a relaxation of tracheal segments.

### Tracheal Histopathology

Twenty-four hours after the last cigarette smoke exposure, the mice were sacrificed. The whole trachea was removed, fixed in 10% formalin, and processed for routine histology in paraffin. Sections were prepared, stained with hematoxylin-eosin and examined under light microscopy. Histology slides were randomly coded, the characteristic lesion features (infiltration of inflammatory cells and tracheal mucous gland hypertrophy) were assessed in a blinded fashion, using a modified scoring system based on those previously described by authors in this field [20-22]. The inflammatory lesion degrees of inflammatory cell infiltration and tracheal mucous gland hypertrophy were both evaluated on a subjective scale of 0, 1, 2, 3, and 4 corresponding to none, mild, moderate, marked, or severe, respectively. The total tracheal inflammation score was defined as the sum of the inflammatory cell infiltration score and the tracheal mucous gland hypertrophy score.

### Statistical analysis

All data are expressed as mean values ± SEM. The concentration-effect curves of agonists were fitted to the Hill equation using an iterative, least square method (GraphPad Prism, San Diego, CA, USA) to provide estimates of maximal contraction (E_max_), maximal relaxation (R_max_) and pEC_50 _values (negative logarithm of the concentration that produces 50% of the maximal effect). Two-way analysis of variance (ANOVA) with Dunnett's test post-test was used for comparisons between all treatment groups. *p *< 0.05 is considered as statistically significant. The comparison of histology scores was analyzed by the Mann-Whitney test. The n equals the number of experimental animals.

## Results

### Tracheal segment hyperresponsiveness to potassium

**Figure 1:**
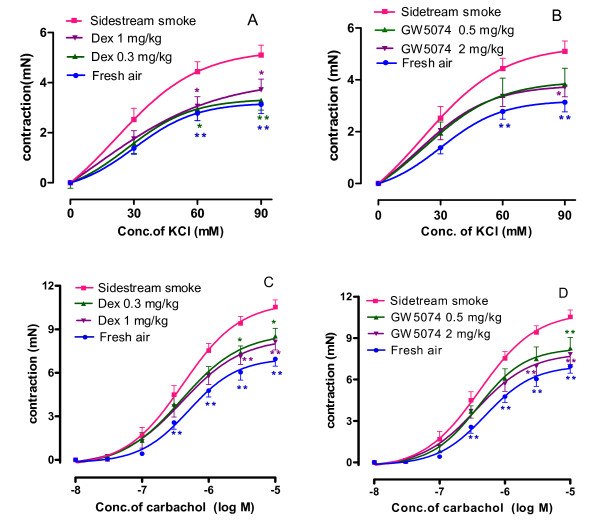
**Effect of dexamethasone (A and C) and GW5074 (B and D) on the concentration-contractile curves of the trachea segments isolated from the sidestream smoke exposed mice induced by potassium chloride (KCl) and by carbachol.** Results are expressed as the mean ± SEM, n = six or seven animals/group, *p < 0.05 and **p < 0.01 vs. sidestream smoke exposure group.

The viability and general contractility of the trachea ring segments from the sidestream smoke exposure group, the fresh air group, dexamethasone plus sidestream smoke exposure groups and GW5074 plus sidestream smoke exposure groups were examined by their contractile responses to a cumulative concentration of potassium chloride. The potassium induced a concentration-dependent contraction of the tracheal ring segments isolated from the fresh air group (Figure 1). The sidestream smoke exposure caused a significant increase in the contraction and shifted the concentration-contraction curves to the left with an increased E_max_ of 5.51 ± 0.46 mN (Figure 1, Table 1), compared with the fresh air group. Treatment of mice with either dose of dexamethasone (0.3 mg/kg or 1 mg/kg) attenuated the potassium-induced contraction of tracheal ring segments in sidestream smoke exposed mice and shifted the concentration-contraction curves to the right with a decreased E_max_ of 3.50 ± 0.45 mN and 3.94 ± 0.52 mN, respectively (Table 1, Figure 1A). The contraction induced by potassium was also significantly decreased by treatment with either dose of GW5074 (0.5 mg/kg or 2 mg/kg) compared with the sidestream smoke exposure group, which had a decreased E_max_ (Table 1, Figure 1B).

**Table 1:** The E_max _and pEC_50 _of the concentration-contractile curves of the trachea segments isolated from the sidestream smoke-exposed mice induced by potassium chloride, carbachol and endothelin-1

			E_max _(mN)			pEC_50_		
		
Group	dose (mg/kg)	n	Potassium	Carbachol	Endothelin-1	Potassium	Carbachol	Endothelin-1
Fresh air	-	7	3.56 ± 0.41^†^	7.01 ± 0.09^†^	3.34 ± 0.03^†^	1.73 ± 0.08	6.30 ± 0.01^†^	7.87 ± 0.01^†^
Smoke	-	7	5.51 ± 0.46	10.87 ± 0.09	5.53 ± 0.04	2.00 ± 0.18	6.39 ± 0.01	7.97 ± 0.01
Dex	0.3	6	3.50 ± 0.45^†^	8.75 ± 0.13^†^	3.94 ± 0.06^‡^	2.02 ± 0.15	6.41 ± 0.02	7.82 ± 0.02
Dex	1.0	6	3.94 ± 0.52*	8.38 ± 0.11^†+^	4.06 ± 0.14^†^	1.98 ± 0.13	6.40 ± 0.02	7.77 ± 0.04
GW5074	0.5	6	4.17 ± 0.66	8.27 ± 0.10^†^	4.12 ± 0.06^†^	1.81 ± 0.10	6.41 ± 0.02	7.80 ± 0.02
GW5074	2.0	6	3.99 ± 0.37*	7.92 ± 0.11^†+^	3.42 ± 0.04^†#^	1.93 ± 0.09	6.44 ± 0.02	7.83 ± 0.01

Data are expressed as the means (SEM). * *p *< 0.05, ^† ^*p *< 0.01, compared with the sidestream smoke-exposed group; ^+ ^*p *< 0.05, ^# ^*p *< 0.01 compared with the low dosage group. E_max_, maximal contraction; pEC_50_, negative logarithm of the agonist concentration that produces 50% of the maximal effect; Dex, dexamethasone.

### Tracheal segment hyperresponsiveness to carbachol

Carbachol, a muscarinic receptor agonist, induced concentration-dependent contractile responses in tracheal segments isolated from the fresh air group. Sidestream smoke exposure resulted in a markedly enhanced contraction and shifted the concentration-contractile curves of the tracheal segments to the left with an increased E_max_ of 10.87 ± 0.09 mN (Table 1, Figure 1C, 1D), compared with tracheal segments of mice exposed to fresh air. Treatment of mice with either dose of dexamethasone (0.3 mg/kg and 1 mg/kg) attenuated the contraction of the tracheal ring segments induced by carbachol in the sidestream smoke exposed mice and shifted the concentration-contraction curves to the right with a decreased E_max_ of 8.75 ± 0.13 mN and 8.38 ± 0.11 mN (*p *< 0.01)(Figure 1C), respectively. Treatment of mice with either dose of GW5074 (0.5 mg/kg or 2 mg/kg) produced similar results as dexamethasone with a reduction in the contractile responses and a decreased E_max_ of 8.27 ± 0.10 mN and 7.92 ± 0.11 mN (*p *< 0.01), respectively (Table 1, Figure 1D), compared with the sidestream smoke exposure group. Moreover, there are statistical differences in the E_max _values in response to carbachol between the two doses of dexamethasone (0.3 vs. 1.0 mg/kg; *p *< 0.05) and between the two doses of GW5074 (0.5 mg/kg vs. 2 mg/kg; *p *< 0.05), which suggests that the suppressive effect is dose-dependent.

### Tracheal segment responsiveness to sarafotoxin 6c

**Figure 2:**
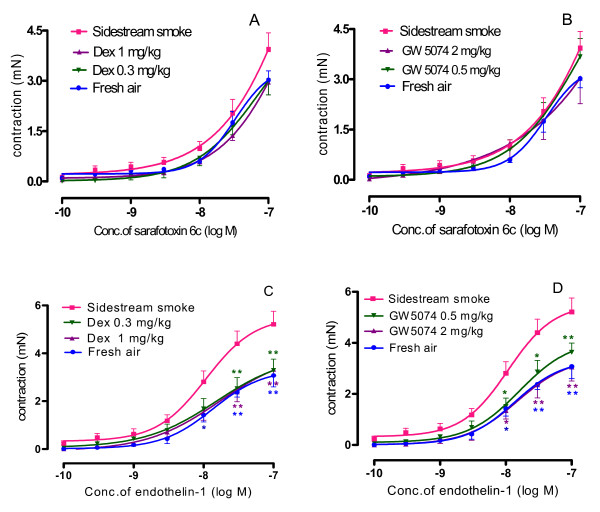
**Effect of dexamethasone (A and C) and GW5074 (B and D) on the concentration-contractile curves of the trachea segments isolated from the sidestream smoke exposed mice induced by sarafotoxin 6c and by endothelin-1.** Results are expressed as the mean ± SEM, n = six or seven animals/group.

Sarafotoxin 6c, a specific agonist of the endothelin ET_B _receptor, caused concentration-dependent contractile responses in all of the mouse tracheal segments from the sidestream smoke exposure group, fresh air group, dexamethasone (0.3 mg/kg, 1 mg/kg) plus sidestream smoke exposure groups and GW5074 (0.5 mg/kg, 2 mg/kg) plus sidestream smoke exposure groups. However, the airway contraction in response to sarafotoxin 6c showed no significant differences among these groups (Figure 2A, 2B). Although at the 1 × 10^-7 ^M dose of sarafotoxin 6c could get a maximal contractive effect in the control group (fresh air exposure), its curve in the smoke-exposed group was incomplete (Figure 2A, 2B). This suggests an enhanced potency of sarofotoxin in the airway after sidestream smoke exposure.

### Tracheal segment hyperresponsiveness to endothelin-1

As described in the methods, the sarafotoxin 6c concentration-effect curve was performed first and the segments remained in contact with sarafotoxin 6c for more than 1 h before the contraction faded down to the baseline levels, thus it could be considered as a desensitization of the endothelin ET_B _receptor. Then, cumulative administration of endothelin-1, a general agonist for both endothelin ET_A _and ET_B _receptors, was conducted to obtain the concentration-effect curves attributed to the activation of the ET_A _receptor. Figure 2C,2D shows that endothelin-1 induced a concentration-dependent contraction of the tracheal segments isolated from the mice in fresh air group with an E_max_ value of 3.34 ± 0.03 mN. The contraction induced by endothelin-1 on the tracheal segments isolated from the sidestream smoke-exposed mice was markedly enhanced and the concentration-contraction curves were shifted to the left with an increased E_max _of 5.53 ± 0.04 mN (*p *< 0.01), compared to the fresh air exposed group. Dexamethasone (0.3 mg/kg, 1 mg/kg) or GW5074 (0.5 mg/kg, 2 mg/kg) administration attenuated the contraction induced by endothelin-1 on the tracheal segments isolated from the sidestream smoke exposed mice with a decreased E_max _of 3.94 ± 0.06 mN, 4.06 ± 0.14 mN, 4.12 ± 0.06 mN and 3.42 ± 0.04 mN, respectively (Table 1, Figure 2C, 2D). There was a statistical difference (*p *< 0.01) in the E_max _values between the mice administered the 0.5 mg/kg and 2 mg/kg doses of GW5074, which suggests a dose-dependent effect.

### Effects on tracheal segment relaxation induced by isoprenaline

**Figure 3:**
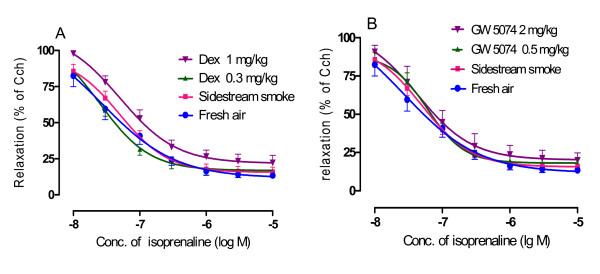
**Effect of dexamethasone (A) and GW5074 (B) on the concentration-relaxation curves induced by isoprenaline in the trachea segments isolated from the sidestream smoke exposed mice, which were pre-contracted with carbachol (Cch) 2 × 10^-7 ^M.** Results are the percent of relaxation induced by isoprenaline after pre-contraction with carbachol and are expressed as the mean ± SEM. n = six or seven animals/group, *p < 0.05 and **p < 0.01 vs. sidestream smoke exposure group.

Airway hyperresponsiveness can be manifested as a response to both increases in the receptors that mediate airway constriction and decreases in the receptors that mediate airway dilatation. β-adrenoceptor is the most important receptor that mediates airway dilatation. In the present study, we investigated the effect of sidestream smoke on the dilatation function of β-adrenoceptor and the effect of GW5074 and dexamethasone. A sustained contraction of the tracheal segments was obtained by carbachol 2 × 10^-7 ^M. Subsequently, cumulative administration of the β-adrenoceptor agonist, isoprenaline, induced a concentration-dependent relaxation of all of the segments of the mouse trachea isolated from the sidestream smoke exposure group, fresh air group, dexamethasone (0.3 mg/kg, 1 mg/kg) plus sidestream smoke exposure group and GW5074 (0.5 mg/kg, 2 mg/kg) plus sidestream smoke exposure group. A significant difference in the concentration-relaxation curves was not observed among these groups (Figure 3).

### Effects on tracheal pathology

**Figure 4:**
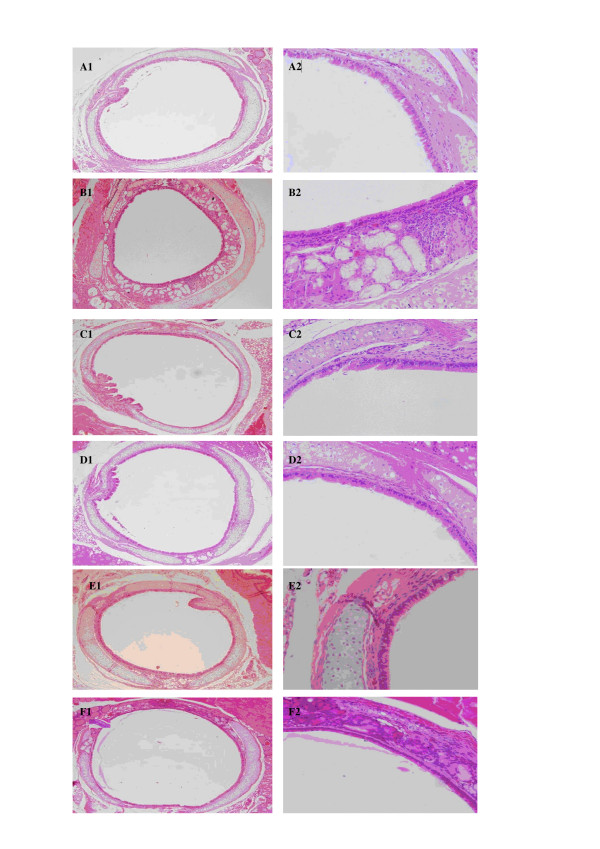
**Effect of dexamethasone and GW5074 on the tracheal pathology of mice exposed to passive smoke. **Hematoxylin and eosin-stained tracheal tissue derived from six groups of mice: fresh air group, passive smoke-exposed group, dexamethasone (0.3 mg/kg, 1 mg/kg) plus passive smoke-exposed groups and GW5074 (0.5 mg/kg, 2 mg/kg) plus passive smoke-exposed groups. Inflammatory cells and tracheal mucous gland hypertrophy were not found in the fresh air group (A1: ×100 and A2: ×400). There were many infiltrated inflammatory cells and mucous gland hypertrophy in the tracheas of the passive smoke-exposed group (B1: ×100 and B2: ×400). The infiltration of inflammatory cells and tracheal mucous gland hypertrophy were decreased in both the 1 mg/kg (C1: ×100 and C2: ×400) and the 0.3 mg/kg (D1: ×100 and D2: ×400) dexamethasone groups and both the 2 mg/kg (E1: ×100 and E2: ×400) and the 0.5 mg/kg (F1: ×100 and F2: ×400) GW5074 groups, compared with the passive smoke-exposed group.

Inflammatory cells were infiltrated into the tracheal smooth muscle layer in the sidestream smoke exposure mice and tracheal mucous gland hypertrophy could also be observed in these mice, while mice in the fresh air group had no infiltrated inflammatory cells or tracheal mucous gland hypertrophy. Compared to the mice in the fresh air group, there were significantly higher scores in the infiltration of inflammatory cells, tracheal mucous gland hypertrophy and total tracheal inflammation in the mice in the sidestream smoke exposure group. Either dose of dexamethasone (0.3 mg/kg or 1 mg/kg) significantly decreased the inflammatory cells infiltration, tracheal mucous gland hypertrophy and the total tracheal inflammation induced by sidestream smoke exposure. Similar results were obtained by treating the mice with two doses of GW5074 (0.5 mg/kg or 2 mg/kg). There were statistical differences in the total scores between the doses of dexamethasone (0.3 and 1.0 mg/kg), and between the doses of GW5074 (0.5 mg/kg and 2 mg/kg), suggesting there is a dose-dependent effect of dexamethasone and GW5074 on airway inflammatory lesions (Table 2, Figure 4).

**Table 2:** The effects of dexamethasone and GW5074 on inflammatory lesions of the trachea segments isolated from the sidestream smoke-exposed mice

Group	dose (mg/kg)	n	inflammatory cells infiltration scores	tracheal mucous gland hypertrophy scores	Total scores
Fresh air	-	7	0.00 ± 0.00^†^	0.00 ± 0.00^†^	0.00 ± 0.00^†^
Smoke	-	7	3.00 ± 0.31	3.14 ± 0.26	6.14 ± 0.40
Dex	0.3	7	1.57 ± 0.20^†^	1.71 ± 0.29^†^	3.29 ± 0.29^†^
Dex	1.0	7	1.14 ± 0.14^†^	1.29 ± 0.18^†^	2.43 ± 0.20^†#^
GW5074	0.5	7	2.00 ± 0.22*	2.14 ± 0.34*	4.14 ± 0.40^†^
GW5074	2	7	1.43 ± 0.20^†^	1.57 ± 0.30^†^	3.00 ± 0.31^†#^

Data are expressed as the means (SEM). Dex, dexamethasone * *p *< 0.05, ^† ^*p *< 0.01, compared with the sidestream smoke-exposed group, ^# ^*p *< 0. 05, compared with the low dosage group.

## Discussion

Cigarette smoke exposure induces airway inflammation and subsequent airway hyperresponsiveness [23-25]. The purpose of the present study was to test if the Raf-1 inhibitor, GW5074, and the anti-inflammatory agent, dexamethasone, can suppress the airway hyperreactivity in a mouse model of sidestream smoke exposure. Intraperitoneal administration of the Raf-1 signal pathway inhibitor, GW5074, or the anti-inflammatory drug, dexamethasone, significantly suppressed the hyperresponsiveness of the airway contraction, while the airway epithelium-dependent relaxation was not affected. In addition, sidestream smoke-induced infiltration of inflammatory cells and mucous gland hypertrophy were attenuated by the administration of either GW5074 or dexamethasone. There has been increasing awareness that passive exposure to environmental tobacco smoke increases the incidence of pulmonary diseases [26,27]. G-protein coupled receptor (GPCR)-mediated airway smooth muscle cell contraction and proliferation are the key events in the development and exacerbation of airway hyperresponsiveness [28-32]. Multiple strategies targeting GPCR signaling may be employed to prevent or manage the airway inflammation and subsequent airway hyperresponsiveness [33]. The present study demonstrates that inhibition of Raf-1-mediated inflammatory signaling may provide a new option for treatment of smoking-associated airway hyperresponsiveness.

There is a strong correlation between sidestream smoke exposure and the inflammatory responses. Sidestream smoke induces a dose-response in the systemic inflammatory cytokine production and oxidative stress [34]. Reactive oxygen species from sidestream cigarette smoke can activate redox-sensitive transcription factors, nuclear factor-kappaB (NF-kB), and activator protein-1 (AP-1), which activate the genes of pro-inflammatory mediators, including TNF-α, IL-1β, and IL-6 [35]. In the present study, infiltration of inflammatory cells into the tracheal smooth muscle layer and tracheal mucous glands hypertrophy were observed in the sidestream smoke exposed mice. The Raf-1 inhibitor, GW5074, or the anti-inflammatory drug, dexamethasone, significantly suppressed the airway inflammation and hyperresponsiveness. This agrees well with other reports that glucocorticoids reduce airway hyperreactivity in asthmatic airways [36,37] and diminish airway inflammation [38-40]. Dexmethasone has been demonstrated to inhibit the up-regulation of the GPCR for bradykinin in an *in-vitro *model of chronic airway inflammation [5]. In previous reports, we have demonstrated [4,6] that activation of intracellular MAPK inflammatory signal transduction pathways are responsible for alteration of the GPCR for bradykinin in airway smooth muscle cells. Raf-1 (C-Raf) is the most widely expressed and considered to be the key protein kinase in the MAPK signal transduction cascade [7]. The Raf-1 inhibitor, GW5074, and the anti-inflammatory drug, dexamethasone, significantly attenuated the sidestream smoke-induced airway inflammation and hyper-responsiveness, suggesting that in the present study, sidestream smoke induced pro-inflammatory responses in mouse tracheas are corticosteroid-sensitive. Raf-1-mediated inflammatory signaling plays a key role in the airway inflammation and hyper-responsiveness.

The contraction evoked by potassium chloride in airway smooth muscle is due to a voltage-dependent Ca^2+ ^influx activation of the Rho/Rho-associated kinase signaling pathway [41]. The closure of the Ca^2+^-dependent K^+ ^channels (BK_Ca_) could increase the mouse tracheal smooth muscle sensitivity to potassium chloride, while the inhibition of the voltage-dependent Ca^2+ ^channels could attenuate the potassium chloride-induced contraction of the mouse trachea [42]. It is reported that dexamethasone can block the protein kinase A-mediated inhibition of Ca^2+^-activated K^+ ^channel (BK_Ca_) activity by modifying a serine/threonine protein phosphatase [43]. Thus, it is possible that the airway hyperresponsiveness to potassium chloride is due to the sidestream smoke exposure, which interferes with the Ca^2+^-activated K^+ ^channel.

## Conclusion

Sidestream smoke induces airway hyperresponsiveness. Inhibition of Raf-1 activity and inflammation suppresses the sidestream smoke exposure effects. Our findings may provide a new pharmacological option for the treatment of smoking-associated airway inflammation and hyperreactivity.

## Competing interests

The authors declare that they have no competing interests.

## Authors' contributions

YL carried out the studies and wrote the first draft of the manuscript. YL and YXC performed the statistical analyses. YXC, CBX and YPZ conceived and designed the study, coordinated and helped to draft and revise the manuscript and contributed key concepts to the study. All authors have read and approved the final manuscript.
